# Impacts of family environment on adolescents’ academic achievement: The role of peer interaction quality and educational expectation gap

**DOI:** 10.3389/fpsyg.2022.911959

**Published:** 2022-09-12

**Authors:** Lie Zhao, Wenlong Zhao

**Affiliations:** School of Humanities and Social Sciences, Xi’an Jiaotong University, Xi’an, China

**Keywords:** family environment, peer interaction quality, educational expectation gap, academic achievement, adolescent

## Abstract

The current study uses a two-wave longitudinal survey to explores the influence mechanism of the family environment on adolescents’ academic achievement. The family environment is measured by parents and children’s reports, including family atmosphere, parent–child interaction, and family rules, to reveal the mediating effect of adolescents’ positive or negative peers between the family environment and academic achievement, and whether the gap between self- and parental educational expectation plays a moderating effect. This study uses the data of the China Education Panel Study (CEPS); the survey samples include 9,449 eighth-grade students (M_age_ = 13.55 years, SD = 0.70), establishing a multilevel moderated mediating effect model. The results showed (1) the family environment and peer interaction quality can positively predict adolescents’ academic achievement. (2) Using the KHB test, peer interaction quality plays a partial mediating role in the process of family environment positively affecting academic achievement, and the mediating ratio is 27.5%. (3) The educational expectation gap moderates the effect of the family environment on academic achievement and also on peer interaction quality. Therefore, from the perspective of environment and important others, to correctly grasp the academic achievement of junior high school students in the process of socialization, it is necessary to recognize that the family environment, peer interaction quality, and educational expectation gap play an important role.

## Introduction

The salient characteristic feature of junior high school students’ academic achievement is the systematic study and participation in various comprehensive practical activities to improve their knowledge and skills in preparation for their future development, including the perception of both students’ self-competence (reading, spelling, language, arithmetic) and school performance (daily ability, writing ability, school satisfaction, calligraphy ability) (Chapman, 1988). Academic achievement in a narrow sense refers to students’ academic performance and course acceptance in school, while academic achievement in a broad sense refers to the knowledge, skills, and cognitive abilities that students acquire through certain teaching and training, both of which reflect the overall learning status and development of students (Sacco, 1997). As the main manifestation of adolescents’ achievements in receiving school education, it is the goal of adolescents’ development in the student period, which is related to the success of adolescents’ future career opportunities. Some scholars focus on adolescents’ cognitive ability and non-cognitive ability (Adams, 2021), prosocial behavior and problem behavior (Karmakar, 2017; Padilla et al., 2018), social development (Walters, 2013), and academic achievement (Zhang et al., 2020) issues; a study found that knowledgeable and successful families are conducive to children’s non-cognitive ability and social development, and a good family atmosphere and a parent–child relationship contribute to the development of physical and mental health (Marcenaro and Lopez, 2017; Obimakinde et al., 2019). Parents who communicate with their children, visit museums, or record daily activities can cultivate children’s information literacy, improve math and reading scores, and directly stimulate cognitive development (Sibley and Dearing, 2014; Choe, 2020). Moreover, family socioeconomic status (SES), related developmental resources (including parental support, expectations, and reading resources), and students’ individual reading motivation (including reading engagement and reading confidence) also affect adolescents’ learning outcomes (including academic achievement, school grades, and reading competence) (Mudrak et al., 2020).

With the increase in communication time between adolescent students and their peers, they gradually break away from their families to participate in peer interaction; peers have become another major field affecting their development (Criss et al., 2016). The interpersonal relationship established by teenagers in the school field plays an essential role in their behavior development, cognitive ability, and academic performance, especially adolescents’ academic engagement or learning performance is influenced by friendship quality (Sebanc et al., 2016), friends’ gain and loss (Lessard and Juvonen, 2018), and peer personality (Golsteyn et al., 2021) factors in peer groups. Reviewing the research on the influence of family and important others on students’ achievement, there are two main points of view: On the one hand, according to American psychologist Harris’ group socialization development theory, parents and peers are the main objects for adolescents to realize social dependence, and they advocate that individual development (physical and mental development, and academic performance) is affected by the two ‘independent systems’ inside and outside the family (Harris, 1995). On the other hand, some scholars expressed that the influence of family members and peer groups on adolescents’ academic achievement was a kind of ‘mutual compensation’ (Fukuoka and Hashimoto, 1997). Although considerable research has involved single factors in the family environment (including socioeconomic status, parental autonomy support, and parental involvement in education) (Joussemet et al., 2005; Vasquez et al., 2016; Froiland and Worrell, 2017; Mudrak et al., 2020), important others, including teacher autonomy support, learning competition among students, and positive or negative learning behaviors of classmates, affect academic achievement (Diseth and Samdal, 2014; Li et al., 2022; Qiu and Chai, 2022). A small number of studies have also introduced emotion regulation, adaptive competencies, and sense of autonomy at the individual level as mediating variables, and these influencing processes are different due to different grades and genders (Liew et al., 2014; Qiu and Chai, 2022). However, from the beginning of junior high school, parents pay more attention to students’ academic achievement, and the time of students getting along with their peers in school is also significantly increased; much less is known about the role of peer relationships in the impact of family environment on academic achievement, and at the middle school stage, differences in educational expectations between parents and adolescents are constantly changing, which is particularly reflected in Chinese students. Therefore, this study is based on the academic achievement of Chinese adolescents; it is necessary to further study the internal relationship between the family environment and academic achievement by introducing the quality of peer interaction as a mediator and the factors of the gap between self- and parental educational expectations. This study seeks intervention measures from the factors of the quality of peer interaction and educational expectation gap, which will provide new ideas for improving the academic achievement of Chinese adolescents.

## Literature review and hypotheses

### Family environment and academic achievement

The family environment is the sum of physical and psychological conditions, which carries the development of individual personality and behavior, among which family relations and parent–child interaction are its important components, affecting children’s academic achievement, character quality, and the expression of psychological modeling functions (Wilder, 2014; Krauss et al., 2020). According to the family systems theory, the family is composed of several subsystems, which are interconnected and mutually constrained to make the whole family function well, and the better the coordination of the family system, the better the psychological shape and academic performance of the members (Miller et al., 1985). Leung and Shek (2016) divided family functioning into five dimensions: family members’ relationship, communication and adaptation, conflict and harmony, parental attention, and parental control; in other words, the more harmonious family functioning is, the higher the self-rated family environment score is, emphasizing that family cohesion and harmonious parent–child relationship can promote adolescents’ physical and mental development. Findings of related scholars’ research on the impact of parental participation on children’s academic performance, development skills, and social behavior are given as follows (McCormick et al., 2013; Karmakar, 2017; Boonk et al., 2018): Parents’ educational level directly affects their children’s reading comprehension and math achievement; among them, the influence of mothers’ education level will be more lasting. Parents’ active participation in education has a significant effect on children’s academic achievement, educational achievement, and mental health, in particular parents’ support for the educational process, the cultivation of extracurricular interest, and the guidance of homework have a strong positive effect on the academic performance of adolescents (Wang and Sheikh, 2014; Benner et al., 2016). The family investment theory explains the effect of the family socioeconomic status on academic achievement (Duleep, 1998). Parents with high socioeconomic status will invest more in their children’s education (parents’ attention, support, and investment), and their children‘s academic achievement will be better (Mudrak et al., 2020; Poon, 2020; Zhang et al., 2020). At the same time, perceived positive emotional expression in the family, daily communication, rule-making, and conflicting parental relationships have varying degrees of impact on adolescents’ behavioral tendencies (learning method and problem behavior) and academic achievement (social science, reading, language, and natural science scores) (Ghazarian and Buehler, 2010; Veas et al., 2019). It can be seen that previous studies mainly focus on the influence of factors from one aspect: family on children’s academic achievement. In addition, while promoting the smooth development of family education, recognizing the characteristics of the cognitive level and social competence of students at the junior high school stage, students’ academic achievements in a broad sense are affected by factors such as family atmosphere, parent–child interaction, and family rules. As Bronfenbrenner emphasized family as a microsystem that directly affects individual development (Bronfenbrenner, 1986), it serves as an educational ground for children’s symbolic values, sense of honor and disgrace, lifestyle, and various action strategies. It is further speculated that the score of the family environment generated by a family atmosphere, parent–child interaction, and family rules will have a direct effect on children’s academic achievement.

### Peer interaction quality and academic achievement

Students in junior high school travel between home and school, with alternating contact with parents and peers, and it is a process of gradual stabilization and continuous cognitive reproduction. In a diverse school, students tend to view themselves by the preferences or standards of their peer group, which subconsciously affects the acquisition of social values and the completion of their studies. In Coleman’s book “*The Adolescent Society*,” he points out that “teens suffering from rejection from peers is almost equivalent to being rejected by their parents” (Coleman, 1961). Combined with the peer group effect theory, peer group interaction conveys social norms, values, knowledge, and skills, and positive or negative peer relationships affect the learning attitude, self-expectation, and cognitive development of the participants (Winkler, 1975). Academic interaction between students in the classroom and the average score are all related to learning performance (math scores); forming a learning group can increase the possibility of cooperating to complete homework and enhance learning interest (Carman and Zhang, 2012). Comparing students in the classroom with their peers in the living environment and interaction with roommates in the informal environment have a stronger impact on academic performance (Jain and Kapoor, 2015; Fang and Wan, 2020). In essence, the structure of the peer network (quality, scale, heterogeneity, and cohesion) and students’ learning behavior (positive and negative) have an effect on students’ academic achievement (Berthelon et al., 2019; Qiu and Chai, 2022). For example, with diligent and dedicated classmates, the higher the quality of peer interaction (more positive peers and less negative peers in peer interaction), the better the results in subsequent learning (Golsteyn et al., 2021). In addition, in a better school, this peer effect will be amplified accordingly, that is, in a better school environment, students can interact with better peers, and the quality of making friends will be higher. They supervise each other in learning, and their academic performance will be better (Wang et al., 2021). Previous studies have not taken the quality of adolescents’ peer interaction as an important variable for research. Therefore, based on the quality of peer interaction in adolescents, that is, the “negative” or “positive” behavior of friends will affect their external performance and internal cognition, it is inferred that the more positive the quality of peer interaction, the more conducive to higher academic achievement.

### Mediating effect of peer interaction quality

The social development of students in adolescence is crucial. As adolescents are gradually fleeing from getting along with their parents to making new friends, it is predicted that the effect of peers in the group on the social action or mentality structure of adolescents is increasing (Brown et al., 1993); peers play an important role in academic achievement. Parents and peers are the main objects for adolescents to rely on and complain to, and both of them play an important role in the process of individual socialization. Sociologists Hartup and Stevens (1997) proposed two different types of interpersonal relationships, vertical and horizontal, for children. Vertical relationship refers to the relationship between children and adults, such as the parent–child relationship and teacher–student relationship, which are complementary and provide children with a safety guarantee and a learning environment. Horizontal relationship refers to the peer relationship with the level of self-development, which has the function of providing physical and mental development and interaction for children. Therefore, students lack the support of parents and peer friendship and are prone to depression, resulting in academic waste, and it is prone to depression, resulting in academic abandonment. Research on the family environment (family social capital and parenting style), peer interaction, and adolescents’ academic achievement, there are two types of views: First, family and peer influence on adolescents is “independent.” According to Harris’ group socialization development theory (Harris, 1995): individuals acquire two independent behavioral systems inside and outside the family—the effect of family on children’s socialization is weakening, while the influence of peer groups in schools is increasing; for example, family education resources and parents’ SES have direct effects on adolescents’ math achievement and problem-solving ability (Long and Pang, 2016; Wang et al., 2021), and peer friendship quality and friends’ gain and loss predict adolescents’ learning engagement and academic achievement (Sebanc et al., 2016; Lessard and Juvonen, 2018)—getting along with friends who study well and live actively influences their initial study and helps in getting better grades. Second, the influence of peers and family on adolescents is a “complementary” view (Fukuoka and Hashimoto, 1997), that is, peer interaction transmits the effect of family environment on adolescents’ academic performance, compared with childhood, at the adolescent stage, parent education and parent-child interaction no longer meet their needs, but gradually extend to seeking support or help among peers, and peer interaction and family environment together influence adolescents’ growth. The family environment (parenting style, behavior supervision, and emotional intervention) plays a decisive role in the quality of peer interaction among adolescents; for example, parents supervise their children’s home time, places to go out, friend interactions, and homework completion, which would increase children’s exposure to peers with positive learning behaviors (Deutsch et al., 2012; DeAnna, 2016), which indicates that parent–child communication and parental educational involvement influence children’s interpersonal interactions. At the same time, the peer network structure, friend quality, and personality orientation also affect students’ academic achievement (Berthelon et al., 2019; Golsteyn et al., 2021). Based on the available research, a more superior family environment may have a positive effect on students’ academic achievement by increasing their peer interaction quality.

### Moderating effect of educational expectation gap

Educational expectation is based on one’s cognitive ability, realistic conditions, and parents’ expectation of children or adolescents’ academic achievements in their future (Wang and Benner, 2014). It belongs to a category of social cognition, including the sender and the expected. When the two are the same individual, it is called “self-education expectation,” and when the expectation is sender by parents and the expectation is expected by children, it is called “parental education expectation” (Wang and Benner, 2014; Castro et al., 2015). The identity control theory points out (Peter, 1991) that parental education expectation is seen as a reflective evaluation of important others, and it is an important type of social environment information input; self-education expectation is regarded as an individual’s identification standard of the current social role. When the two are inconsistent, individuals will have a sense of stress, which even leads to psychological distress and affects development, and is closely related to individual intrinsic motivation (Moe, 2016). When parents’ educational expectation is moderate, it is conducive to the cultivation of children’s social value and the shaping of healthy personality, while when parents’ expectation is much higher than their children’s self-expectation, it will make the goal impossible to achieve, resulting in tense parent–child relationship and weariness of learning, which will harm academic achievement (Marcenaro and Lopez, 2017; Lv et al., 2018). On the one hand, children’s reading and math scores are related to net household assets; the higher the SES of parents, the higher the educational expectation for their children, providing a quality educational environment to ensure that children have good supportive resources. Parents’ higher education expectation or lower self-education expectation moderates this effect (Zhan, 2006; Zhang et al., 2011). The higher parental expectation and short-term educational expectation in junior high school have a lasting positive effect on children’s academic performance (school achievement, test scores, and academic completion) (Yamamoto and Holloway, 2010). In fact, in the study of parents’ expectations and their children’s academic achievement (reading achievement and academic achievement), parents’ expectation of their children’s study is consistent with their expectation of self-education, which can better improve their social cognitive ability (Phillipson and Phillipson, 2012; John and Bierman, 2017). It shows that the educational expectation gap between children and parents will moderate the impact of the family environment on academic achievement.

On the other hand, according to the analysis of the British Household Panel Survey (BHPS) data, children grow up under the emotional education of their parents and have a better experience of happiness, making friends with active companions, less likely to fight, smoke, or take drugs (Chan and Koo, 2011). In the study of 497 Dutch adolescents (13 years) from exposure to negative peers to crime, parents’ excessive restrictions on their children’s friends hinders their ability to develop autonomously and increases the risk of having bad peers (Keijsers et al., 2012). Parents with higher SES have a higher expectation for their children, which accordingly enlarges the negative effects of problem peers. This process reflects that parents’ expectations, or parenting styles moderate the impact of family socioeconomic status on deviant peers (Forgatch et al., 2016; Valdivia and Castello, 2020). Reviewing the research on the differences between children’s self-education expectation and perceived parents’ educational expectation, parents’ educational expectation is not always consistent with that of their offspring; both have different perceptions of future educational goals, which is universal (Rutherford, 2015). When there is a large gap between parents’ educational expectation and self-educational expectation, the educational expectation gap affects the quality, scale, and structure of children’s peer relationship. Based on this, this study introduces the concept of the “educational expectation gap,” speculating that the intergenerational educational expectation gap plays a moderating role between the family environment, peer interaction quality, and academic achievement.

### Present study and hypotheses

**FIGURE 1:**
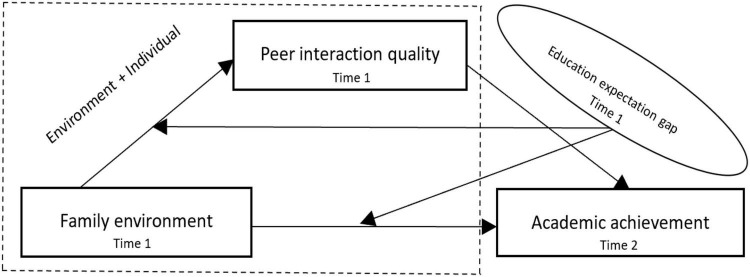
Moderated mediation model.

Previous studies reported the relationship between the family environment and academic achievement (Benner et al., 2016; Boonk et al., 2018; Veas et al., 2019; Mudrak et al., 2020) and introduced the factors of parental education expectation, self-education expectation, and peer interaction (Phillipson and Phillipson, 2012; Sebanc et al., 2016; Lessard and Juvonen, 2018; Qiu and Chai, 2022). Among them, most studies regard parental education expectation and self-education expectation as separate variables to examine the effect of the family environment on academic achievement (Marcenaro and Lopez, 2017; Lv et al., 2018), and some scholars also studied the influence of the peer network structure, friendship quality, and personality orientation on adolescents’ academic achievement (Berthelon et al., 2019; Golsteyn et al., 2021). Parents supervise their children’s home time, places to go out, friend interactions, and homework completion, which would increase children’s exposure to peers with positive learning behaviors, which may have a positive impact on children’s academic achievement (Deutsch et al., 2012; DeAnna, 2016). However, few studies have emphasized the impact of the family environment and peer interaction quality on adolescents’ academic achievement and the role of the gap between parents’ and children’s educational expectations in this process, in particular the study of adolescents who are in the middle school stage and have high expectations for parental education and more contacts with peers. Therefore, according to the ecosystem theory (Bronfenbrenner, 1986), the environment in which human beings live consists of four systems: microsystem, mesosystem, external system, and macrosystem, among which the microsystem refers to the way of activity, role patterns, and interpersonal relationship patterns of individuals in a particular environment; the way of behavior that promotes or inhibits individuals in that environment; and the interaction between individuals and that environment, which directly affects human cognitive ability, social development, and academic achievement; that is, the family environment and peer interactions are important microsystems of the individuals’ lives. This study constructs an analytical framework of significant others embedded in the family environment and then combines the peer group effect (Winkler, 1975) and the identity control theory (Peter, 1991), which emphasize the differences from significant others, self, and other identity criteria, to explore the role of the quality of peer interactions and educational expectation gap of adolescents in the microsystem in the impact of the family environment on academic achievement. Based on available research results, this study proposed the following hypotheses and a moderated mediation model (see Figure 1).

**Hypothesis 1 (H1)**: Family environment will have a direct effect on adolescents’ academic achievement.

**Hypothesis 2 (H2)**: Peer interaction quality will have a direct effect on adolescents’ academic achievement.

**Hypothesis 3 (H3)**: Peer interaction quality will play a mediating role between family environment and adolescents’ academic achievement.

**Hypothesis 4a (H4a)**: Educational expectation gap will moderate the effect of family environment on adolescents’ academic achievement.

**Hypothesis 4b (H4b)**: Educational expectation gap will moderate the effect of family environment and adolescents’ peer interaction quality.

## Materials and methods

### Research data

The data for this study are from the nationally representative “China Education Panel Study” (CEPS) implemented by the China Survey and Data Center of the Renmin University of China. The survey involves students, schools, and districts of multilevel characteristic variables, using a stratified multistage, probability, and scale proportional (PPS) sampling method. A total of 438 classes were randomly selected from 112 schools of 28 county-level units, and all the students in the selected class were investigated, in the baseline survey in 2015. A total of 10,279 junior middle school seventh-grade students were present after data merging and missing values filling, and 9,449 eighth-grade students successfully tracked in the 2016 follow-up survey are used as effective samples.

### Variables and measurements

#### Academic achievement

Combining the narrow and broad definitions of academic achievement (Sacco, 1997), it refers to students’ academic performance and course acceptance at school, as well as knowledge, skills, and cognitive abilities acquired through certain teaching and training. The academic achievement of this study is measured by three indicators: students’ cognitive ability, test scores, and the acceptance ability of the main courses (Chinese, Math, and English). The project team designed a cognitive ability scale, which includes 22 items in three dimensions of language, graphics, and computing and logic, to measure students’ logical thinking and problem-solving ability. Each student’s score is used to measure cognitive ability. Referring to the academic achievement index (Li et al., 2022), test scores are the total scores of students’ Chinese, math, and English midterm examinations. The ability to accept the main course is measured by asking students whether “is it hard to learn at present?” in the three courses of Chinese, math, and English, and each question corresponds to four options, with 1 representing “special effort” and 4 representing “no effort.” The scores of the three question items were summed to generate a continuous variable with a value range of 3–12. The higher the score, the easier the learning process experience. Based on the fact that academic achievement in this study includes three dimensions of cognition, objectivity, and subjectivity; the common factors of these three dimensions are extracted through exploratory factor analysis (EFA). The first factor is 1.63, and the second factor is 0.75 (1.63/0.75 = 2.17, 2.17 < 3), indicating that the constructed academic achievement is multidimensional. At the same time, considering that in the item response theory (IRT) model, the respondents’ response to the project (the probability of right answer) and their potential (psychological traits) have a certain connection and need to meet the measurement is a one-dimensional premise assumption (Lord and Wingersky, 1984; Reise et al., 1993). Therefore, the method of dimension reduction of academic achievement by the EFA test is better. The results show that the cumulative variance contribution rate is 80.2%, the Kaiser–Meyer–Olkin (KMO) value is 0.714, and the Bartlett sphericity test is significant (*p* < 0.05), and the constructed academic achievement index has less information loss and strong representativeness.

#### Family environment

Based on the Family Environment Scale (Oliver et al., 1988), Family Environment Scale-Chinese Version is revised (Ni et al., 2021), including intimacy, emotional expression, contradiction, independence, success, culture, entertainment, morality, organization, and control; Cronbach’s alpha coefficients of each dimension is between 0.68 and 0.87. Combining with the Effective Pre-school and Primary Education project (EPPE), the scale includes parents’ attitudes and interaction with parents (Eisenstadt, 2010). In this study, 29 items of the family atmosphere (seven items), parent–child interaction (eleven items), and family rules (eleven items) in CEPS were selected to evaluate adolescents’ family environment, using a three-point Likert scale; the higher the total score, the better the family environment, and Cronbach’s alpha coefficient of the questionnaire is 0.84.

#### Peer interaction quality

Using the Reference Friendship Quality Scale (FQS) (Bukowski et al., 1994) and peer relationship measurement (Martina et al., 2020), according to the items included in CEPS, positive peer interaction among students “friends with good grades, hard work, and want to go to college” (three items), and negative peer interaction “absence of class, violation of school discipline, fighting, smoking and drinking, Internet cafes or game hall, early love, dropout” negative peer interaction (seven items) were investigated. All were scored by a three-point Likert scale. Comparing the score of positive peer interaction with the total score of positive and negative peer interaction, the value is a continuous variable; the larger the value, the more positive the peer interaction and the higher the peer quality. We conduct confirmatory factor analysis (CFA) on 10 items; the average variance extracted (AVE) of each second-order factor is between 0.570 and 0.594, which is greater than 0.50, and the composite reliability (CR) is between 0.798 and 0.910, which is greater than 0.70, indicating that the aggregation validity is high. The results of the model showed that Chisq = 1206.813, df = 34, Chisq/df = 35.494, RMSEA = 0.060, RMR = 0.006, GFI = 0.974, CFI = 0.976, TLI = 0.968, indicating that the results of CFA had good fitting indicators, and the resulting peer interaction quality had good stability and fitting degree.

#### Educational expectation gap

Based on the measurement of educational expectation (Rutherford, 2015; Marcenaro and Lopez, 2017), since the general educational expectation of Chinese parents for their children is whether or not they can get into college, in this study, parental education expectation was measured by the parental question “what degree do you want your child to read?”; similarly, students’ self-education expectation was measured by the student question “what degree do you want yourself to read?.” Among the operationalized variables, parental education expectation was divided into two groups: parents want their children to go to college and not to have to go to college. Similarly, students’ self-education expectations were divided into two groups: students who wanted and did not want to get themselves into college. The difference between the former and the latter is used to generate the “educational expectation gap” variable; that is, parental education expectation below or equal to students’ self-expectation is labeled “low educational expectation,” and parental education expectation above students’ self-expectation is labeled “high educational expectation.” The educational expectation gap was converted into a dichotomous variable with a value of 0 or 1.

### Analytical strategy

Considering the heterogeneity between different schools and relatively high homogeneity among students in the same school, we established a multilevel linear model to explore the relationship between adolescents’ academic achievement at the individual and school levels. We used Stata 15.1 software to transform the academic achievement (time 1 and time 2) into the range of 0∼100 by range normalization (Hoffman, 2015; Maslowsky et al., 2015), that is, X’ = (X–X_min_)/(X_max_–X_min_) × 100; eliminate the influence of variation dimension and variation range; and ensure that the estimated results can be compared under the same dimension: (1) Descriptive statistical results were presented, and 4,481 female students (47.4%) and 4,968 male students (52.6%) were included in the follow-up survey. The age ranged from 12 to 18 years old, M_age_ = 13.55 years, SD = 0.70. There were 4,214 singleton students (44.6%) and 5,235 non-singleton students (55.4%). Table 1. (2) The differences in the academic achievement of students with different family backgrounds and the correlation test of core variables were analyzed. (3) The influence of family environment and peer interaction quality in the base period survey (time 1) on the academic achievement tracked (time 2), and the role of educational expectation gap in it were also analyzed; if *p* < 0.05, regression is considered to be important. Academic achievement is measured by a lag phase of data, which can solve the endogenous problem and predict current academic achievement from the past environment or other factors. It has a clearer causal logic relationship (Hoffman, 2015). The main study steps are as follows: First, a null model M0 with a random intercept but no explanatory variable is estimated to explore the total difference in academic achievement, which is decomposed into the difference between students and schools, and establish a school fixed effect model (Bryk and Raudenbush, 1987). Second, model M1 tests the effect of the family environment. Third, model M2 tests explain differences in the peer interaction quality on academic achievement. Fourth, model M3 also adds the effects of the family environment and peer interaction quality prediction on academic achievement. Model M4 takes the sum of base period data and tracking data (academic achievement) as the dependent variable to test the robustness of the model. Finally, models M5, M6, and M7 are constructed to test whether peer interaction quality plays a mediating role between the family environment and academic achievement. The mediating effect test usually includes the Sobel (1982) test and stepwise method (Baron and Kenny, 1986); these two methods require the assumption that the product term variables formed by the two methods have normal distribution, resulting in low test power on the test coefficient items in turn. In smaller samples, the bias-corrected bootstrap often reduces the error more than other methods (Hayes and Scharkow, 2013). This study explores the effect of the family environment on academic achievement through mediating variables and uses KHB method to test the effect and size of mediation (Kohler et al., 2011); the method can be any of Regress, Logit, Ologit, Probit, Oprobit, Cloglog, Slogit, Scobit, Rologit, Clogit, Mlogit, Xtlogit, or Xtprobit, and it can be extended to other models. At the same time, we also used the bootstrap repeated test results to further verify that peer interaction quality does play a mediating role. The models M5^ and M6^ are used to verify the moderating effect of the educational expectation gap; if the product of predictor (family environment) and moderator (educational expectation gap) has a significant effect on outcome variables (academic achievement and peer interaction quality) (Baron and Kenny, 1986), it proves that the moderating variable plays a moderating role.

**TABLE 1:** Variables of descriptive statistical results.

Variables			M	SD	Min	Max
**Control variable**	**Individual–level (*N* = 9,449)**					
	Gender (Man = 1)		0.53	0.50	0	1
	Age		13.55	0.70	12	18
	One child (Yes = 1)		0.45	0.50	0	1
	Household registration (Urban = 1)		0.49	0.50	0	1
	Parents’ education (Bachelor degree = 1)		0.20	0.40	0	1
	Father’ s political identity (Party member = 1)		0.16	0.37	0	1
	**School–level (*n* = 112)**					
	School rank (Middle level = 1)		0.81	0.39	0	1
	School type (Public school = 1)		0.94	0.24	0	1
	School location (Central urban = 1)		0.40	0.49	0	1
Major variable	Time 1	Family environment	67.50	8.50	32	87
		Peer interaction quality	48.64	8.09	13	56
		Educational expectation gap (High = 1)	0.17	0.38	0	1
		Academic achievement	63.48	13.46	0	100
	Time 2	Academic achievement	56.02	17.01	0	100

Brackets as the reference group. Academic achievement standardized to 0∼100.

## Results

### Class differences, correlation, and fixed effect test of adolescents’ academic achievement

**FIGURE 2:**
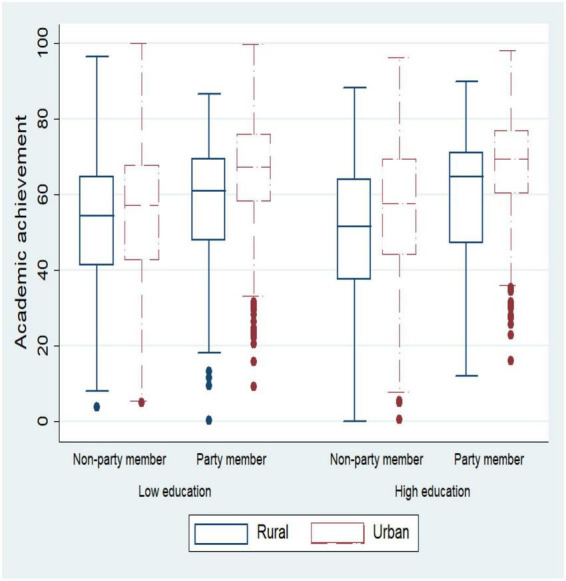
Differences in adolescents’ academic achievement in different households.

**FIGURE 3:**
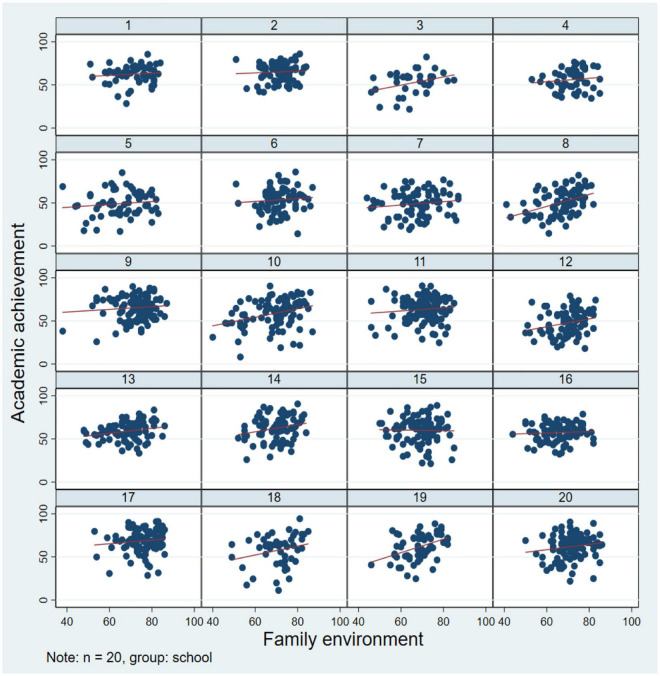
Fixed effect test of different school academic achievements.

Analyzing the differences in adolescent academic achievement across family backgrounds (see Figure 2). Adolescents with high education-level parents, party membership, and household registration in urban areas have good academic achievements. In addition, in order to reveal the factors affecting adolescents’ academic achievement accurately, we tested the correlation of core variables. Academic achievement was positively correlated with the family environment (*r* = 0.31, *p* < 0.05) and peer interaction quality (*r* = 0.39, *p* < 0.05), and the family environment was positively correlated with peer interaction quality (*r* = 0.28, *p* < 0.05). The educational expectation gap was negatively correlated with academic achievement (*r* = −0.15, *p* < 0.05), family environment (*r* = −0.07, *p* < 0.05), and peer interaction quality (*r* = −0.13, *p* < 0.05). At the same time, we selected 20 schools as a small sample, aiming at determining the main effect of each school’s adolescents’ family environment on academic achievement, and measured the different intercepts and slopes in different schools, which confirmed the need to build a fixed effect model (see Figure 3).

### Multilevel regression estimation of adolescents’ academic achievement

As given in Table 2, adolescent academic achievement was considered as the dependent variable and M0 as a null model, and the overall differences in academic achievement are broken down into differences between students and schools. The ICC between groups was 0.319, which shows that it is very suitable to use a multilevel regression model to control for heterogeneity factors between schools and to better estimate the net effect of family and peer-level factors on academic achievement. After M1 controls variables at the individual and school levels, the family environment has a positive impact on academic achievement (β = 0.26, *p* < 0.001); hence, H1 is verified. Similarly, in M2, peer interaction quality has a positive effect on academic achievement (β = 0.47, *p* < 0.001), that is, for every one-unit increase in the quality of peer interaction, adolescents’ academic achievement significantly increases by 47.0%; hence, H2 is verified. In M3, the family environment and peer interaction quality are added to confirm that the coefficients of the multilevel nested model have good stability, and it was found that the coefficient of family environment variables decreased from 26.0% to 19.0% after joining peer interaction quality, and it was still significant. It is necessary to further test the internal mechanism of the family environment and peer interaction quality affecting academic achievement. In M4, the mean value of the dependent variable (academic achievement) of the baseline survey and the follow-up survey is calculated. We built a full model, and the results are consistent with the influence coefficients in M3, showing that the whole research has good robustness.

**TABLE 2:** Multilevel regression estimation results of adolescents’ academic achievement.

	M0	M1	M2	M3	M4 (Robust Test)
**Individual–level**					
Gender		−4.41*** (0.28)	−3.10*** (0.28)	−2.88*** (0.28)	−3.11*** (0.28)
Age		−3.38*** (0.22)	−3.21*** (0.22)	−3.06*** (0.22)	−3.05*** (0.21)
One child		0.91** (0.34)	0.94** (0.34)	0.80** (0.33)	0.77* (0.33)
Household registration		0.15 (0.34)	0.28 (0.33)	0.20 (0.33)	0.26 (0.32)
Parents’ education		3.98*** (0.43)	4.03*** (0.42)	3.57*** (0.42)	3.78*** (0.41)
Father’ s political identity		−0.17 (0.40)	0.11 (0.39)	−0.05 (0.39)	−0.14 (0.38)
**School–level**					
School rank		3.01^+^ (1.69)	2.56 (1.58)	2.16 (1.52)	1.52 (0.99)
School type		1.75 (2.62)	1.39 (2.46)	1.18 (2.36)	0.01 (1.53)
School location		5.60*** (1.43)	5.59*** (1.34)	5.36*** (1.29)	3.21*** (0.84)
Family environment		0.26*** (0.02)		0.19*** (0.02)	0.21*** (0.02)
Peer interaction quality			0.47*** (0.02)	0.43*** (0.02)	0.45*** (0.02)
Educational expectation gap				−3.08*** (0.37)	−3.45*** (0.36)
Constant	55.51*** (0.93)	78.78*** (4.36)	71.44*** (4.11)	60.03*** (4.20)	61.44*** (3.72)
School–level variance	9.71	6.89	6.44	6.19	3.82
Student–level variance	14.19	13.55	13.25	13.14	12.97
ICC	0.319	0.206	0.191	0.182	0.080
Log-likelihood	−38677.11	−38208.30	−37994.39	−37907.77	−37741.10
Observation case	9449	9449	9449	9449	9449
Observation group	112	112	112	112	112

^+^*p* < 0.1, *p < 0.05, **p < 0.01, ***p < 0.001.

### Family environment, peer interaction quality, and academic achievement: Moderated mediation model test

Table 3, in M5, academic achievement as the dependent variable, the total effect of the family environment on academic achievement is 26.0%. In M6, with peer interaction quality as the dependent variable, the effect of family environment on peer interaction quality is 17.0%. In M7, with academic achievement as the dependent variable, the direct effect of adolescents’ family environment on academic achievement is 22.0%, which was 4.0% lower than the total effect (0.26–0.22 = 0.04), and the peer interaction quality has a significant impact on academic achievement (β = 0.42, *p* < 0.001). It shows that peer interaction quality transmits the influence of the family environment on adolescents’ academic achievement, especially the KHB test shows that peer interaction quality plays a partial mediating role in the process of the family environment affecting academic achievement, and the mediating ratio is 27.5%; hence, H3 is verified. Meanwhile, in M5^, the interaction between the family environment and educational expectation gap has a negative significant effect on academic achievement (β = −0.24, *p* < 0.001), and peer interaction quality moderates the effect of the family environment on adolescents’ academic achievement; hence, H4a is verified. In M6^, the interaction between the family environment and educational expectation gap had a negative effect on peer interaction quality (β = −0.07, *p* < 0.01), and peer interaction quality moderates the effect of family environment on peer interaction quality of adolescents; hence, H4b is verified. It shows that in the case of different educational expectations, the influence of the family environment on adolescents’ academic achievement and peer interaction quality is different; compared with the high-education expectation group, the influence of the family environment on adolescents’ academic achievement (β = −0.24, *p* < 0.001) and peer interaction quality (β = −0.07, *p* < 0.01) was weaker than that of the low-education expectation group.

**TABLE 3:** Peer interaction quality and educational expectation gap: Moderated mediation effect test.

	M5	M5^	M6	M6^	M7
	
Control variable	Yes	Yes	Yes	Yes	Yes
Family environment (FH)	0.26*** (0.02)	0.29*** (0.02)	0.17*** (0.01)	0.18*** (0.01)	0.22*** (0.02)
Educational expectation gap (EQ)	−3.70*** (0.38)	12.09*** (3.15)	−1.48*** (0.20)	3.06^+^(1.70)	10.79*** (3.07)
FH × EQ		−0.24*** (0.05)		−0.07** (0.03)	−0.21*** (0.05)
Peer interaction quality (TB)					0.42*** (0.02)
Constant	79.34*** (4.34)	77.02*** (4.34)	45.70*** (2.00)	45.03*** (2.01)	58.10*** (4.22)
School–level variance	6.84	6.84	1.96	1.96	6.19
Student–level variance	13.48	13.46	7.27	7.26	13.12
ICC	0.205	0.205	0.068	0.068	0.182
Log- likelihood	−38160.23	−38147.50	−32255.03	−32251.40	−37897.42
Observation case	9449	9449	9449	9449	9449
Observation group	112	112	112	112	112

^+^*p* < 0.1, **p < 0.01, ***p < 0.001.

**FIGURE 4:**
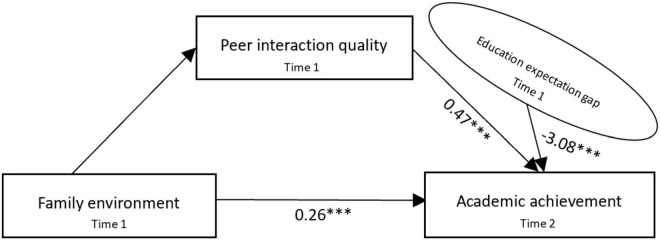
Moderated mediation model (H1 + H2). ***p < 0.001.

**FIGURE 5:**
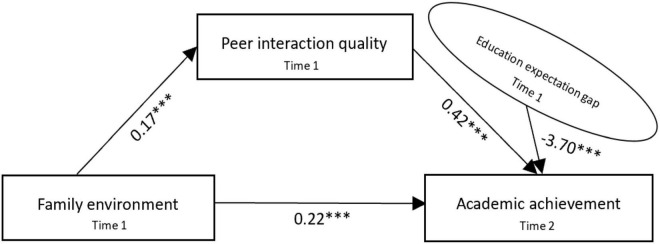
Moderated mediation model (H3). ***p < 0.001.

**FIGURE 6:**
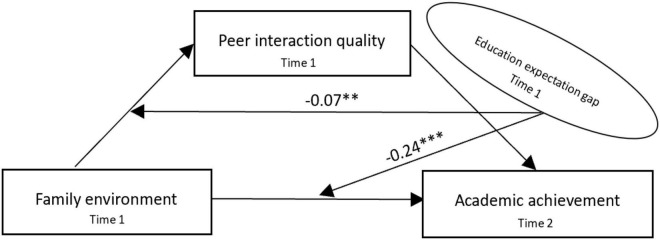
Moderated mediation model (H4a + H4b). **p < 0.01, ***p < 0.001.

According to the estimation results of the models in Tables 2, 3, we further draw the path diagram of the moderated mediation model. Figure 4 shows that the family environment and peer interaction quality have a significant positive effect on adolescents’ academic achievement of 0.26 and 0.47, respectively, which clearly verifies H1 and H2. Figure 5 shows the coefficients of the three paths of family environment → peer interaction quality, peer interaction quality → academic achievement, and family environment → academic achievement are 0.17, 0.42, and 0.22; it is measured that peer interaction quality transmits the effect of the family environment on adolescents’ academic achievement by 27.5% [(0.17 × 0.42)/0.26 = 0.275]. At the same time, bootstrap was used for the mediating test (Hayes and Scharkow, 2013), and the mediating effect of peer interaction quality was tested by repeated sampling for 1000 times using the bootstrap method; the 95% confidence interval (CI) was [0.163, 0.196], which again verified that peer interaction quality plays a mediating role before family environment and academic achievement; hence, it supports H3. Figure 6 shows that the effect of interaction between the family environment and peer interaction quality on academic achievement is −0.24, and the effect of interaction between the family environment and educational expectation gap on peer interaction quality is −0.07, both of which indicate that the higher educational expectation gap will put adolescents’ academic achievement and peer interaction quality at a disadvantage, thus verifying H4a and H4b.

## Discussion

Students in the junior middle school stage are in puberty where their physical and mental development are not yet mature and are easily affected by important others and external environmental factors. Therefore, this study explores the relationship among the family environment, peer interaction quality, educational expectation gap, and adolescents’ academic achievement and further promotes the development of relevant theories. It also has important practical significance to improve adolescents’ academic achievement. On the one hand, this study is based on the ecosystem theory (Bronfenbrenner, 1986), peer group effect theory (Winkler, 1975), and identity control theory (Peter, 1991). Placing adolescents’ academic achievement in a system influenced by the interaction of individual and environment, interactions between peer groups convey social norms and values, as well as parental education expectation is regarded as a reflective evaluation of important others, and there are differences between it and self-education expectation as the standard of individual’s current role orientation. Practical combined with theory, an analytical framework was constructed to study the academic achievement of adolescents, and it was verified that family environment and peer interaction quality play a positive role in academic achievement, which is basically consistent with previous research results (Carman and Zhang, 2012; Boonk et al., 2018; Berthelon et al., 2019; Zhang et al., 2020), greatly expanded the Ecosystem Theory embedded in micro theory (Peer Group Effect, Identity Control) to study the academic achievement of adolescents.

On the other hand, this study also has important practical significance. It makes us understand the mediating role of peer interaction quality (from the influence of important others) between the family environment and academic achievement and enriches the research on the influence of the family environment and important others on academic achievement. It is necessary to pay attention to the influence of the family environment on children’s academic achievement in multiple ways, to create an active family atmosphere, frequent parent–child interaction, and strict family rules and to dynamically understand the quality of children’s peers. At the same time, the influence of peer interaction quality on academic achievement is a double-edged sword. When parents’ education expectation is higher than self-education expectation, it will not only negatively affect adolescents’ academic achievement but also lead to more negative peers; however, the gap between parents’ education expectation and self-education expectation is moderate, which plays a positive role in adolescents’ academic achievement (Zhang et al., 2011; Marcenaro and Lopez, 2017). Therefore, we call on all sectors of society to pay attention to the moderate expectations of parents in the family for their children’s future roles or achievements and avoid pressure caused by too high or too low expectations. This plays an important role in children making positive peers and friends and achieving good academic achievements.

### The mediating effect of peer interaction quality between family environment and academic achievement

The family environment is the external support resource to ensure adolescents’ academic success and the premise of various related factors in the teaching process. It is the initial field of children’s socialization and the carrier of shaping good academic achievements. Through the mediating effect test, the peer interaction quality of adolescents conveys the partial effect of the family environment on academic achievement. The empirical study dialogs with Harris’ group socialization theory and further verifies that peer interaction quality is the link between the family environment and children’s academic achievement (Fukuoka and Hashimoto, 1997). The family environment directly affects the individual’s academic achievement, which is consistent with the results of studies indicating that family socioeconomic status, parents’ attention, support, and investment in children’s education affect their academic performance (Poon, 2020; Zhang et al., 2020). At the same time, the family function also affects group selection and friend composition in individual peer interaction. Interaction with peers exerts a subtle influence on children’s academic achievement and personality shaping, which is consistent with previous studies (Deutsch et al., 2012; DeAnna, 2016). In short, the present study introduced the mediating variable of peer interaction quality, which distinguished it from previous studies that commonly used learning anxiety, learning engagement, sense of autonomy, and parental involvement as mediating variables to build multiple or chained mediation models (Li et al., 2022; Qiu and Chai, 2022), greatly enriches the research on academic achievement, putting children’s academic achievement in the symbolic social living space where the family field and peer network are nested, to form a dynamic field to assist children’s social development and renewal, through peer interaction quality (important others) indirectly affect individual academic achievement, this provides clues and support for further exploring the influence of peer groups on students’ academic achievement.

### The moderating effect of educational expectation gap between family environment, academic achievement, and peer interaction quality

We propose a moderated mediation model based on relevant theories, by examining the role of the educational expectation gap within the family. Parents’ high educational expectations have a negative moderating effect on children’s academic achievement. That is, the model of ‘mother’s actual education wish >self-education wish’ negatively predicts academic performance, while the model of “mother’s actual education wish <self-education wish” positively predicts academic performance (Wang and Benner, 2014). Differences in educational expectations similar to those between parents and children will hinder children’s reading, mathematics, language, and grade point average (GPA) (Rutherford, 2015; Marcenaro and Lopez, 2017). The difference in educational expectations between parents and children is the product of the normal development process of an individual, and it is related to the pressure within the family (family function disintegration, poor family interaction, and poor family cohesion) that can make family members inconsistent (Minuchin, 1985). It may also be the reason for the correlation between the intrinsic motivation of adolescents (including enthusiasm, pleasure, interest, enjoyment, and curiosity) and self-expectation (Moe, 2016). This provides empirical support for the self-discrepancy theory, which points out that there are differences in real self, ideal self, and ought-to self, resulting in an unsatisfactory state, which may lead to depression and affect individual academic development (Higgins, 1987). At the same time, the educational expectation gap moderates the effect of family environment on peer interaction quality, and it is consistent with previous studies which show that parents with low behavior control or high psychological supervision increase children’s chances of contacting poor peers. Peer transmission affects adolescents’ behavior development (Forgatch et al., 2016; Valdivia and Castello, 2020). Based on this, for adolescents with a better family environment, a moderate educational expectation of parents and children can protect peer interaction quality and academic achievement, while excessive educational expectation gap between parents and children will increase the psychological burden of adolescents and have a negative impact on academic achievement. It expands the research of the educational expectation difference between parents and children in the field of individual academic achievement, taking into account the objective environmental factors of the family. We should also include the potential role of important others (parental expectations and peer interaction) in a diversified environment.

### Limitations and future research directions

This study has some limitations and needs to be improved in future research: First, based on the theoretical basis, this work longitudinally studies the influence mechanism of the family environment on academic achievement, which provides empirical support for relevant theoretical viewpoints. However, the self-report in the tracking data may be biased, and experimental research will be used in the next step to obtain more reliable conclusions. Second, the data used are not designed to investigate students’ academic achievements. Future research will design a special questionnaire to collect data to ensure more accurate data information so as to monitor students’ academic development.

In conclusion, this study preliminarily verifies that peer interaction quality plays an intermediary role between the family environment and academic achievement. The educational expectation gap between parents and self within the family moderated the pathways of family environment → peer interaction quality (the first half path), and family environment → academic achievement (the direct path). Using the national-level survey data, rather than limited to a specific area of a small sample survey, a multi-country comparative study is planned for the next step. And further follow-up the factors of achievement motivation, emotional engagement and enthusiasm level of adolescents’ individual learning, the Structural Equation Model (SEM) or Chain Multi-mediary Model will be established to better capture adolescents’ academic achievement jointly from two dimensions: family microsystem and important others (peer interaction quality), enrich and extend the views of relevant theories, to provide practical enlightenment for a more scientific grasp of adolescents’ academic achievement.

## Conclusion

This study uses longitudinal data from a survey of Chinese adolescents. So far, two waves of data have been collected. The research objects were 9,449 eighth-grade students who were successfully tracked, to explore the relationship between adolescents’ family environment (baseline survey) and academic achievement (follow-up survey), and pay special attention to the mediating effect of peer interaction quality between them, and the moderating effect of the gap between self- and parental educational expectations in this process. The results showed that first, the family environment and peer interaction quality can positively predict students’ academic achievement. Second, peer communication quality of adolescents plays a partial mediating role in the process of the family environment positively affecting academic achievement, with a mediating ratio of 27.5%. Third, the educational expectation gap not only moderates the path of the family environment directly influencing academic achievement but also moderates the first half path of the family environment influencing academic achievement through peer interaction quality; that is, the existence of a high educational expectation gap within the family will inhibit the positive effect of the family environment on adolescents’ academic achievement and peer interaction quality.

## Data availability statement

Publicly available datasets were analyzed in this study, this original data can be found here: http://ceps.ruc.edu.cn.

## Ethics statement

The studies involving human participants were reviewed and approved by the Renmin University of China. Written informed consent to participate in this study was provided by the participants’ legal guardian/next of kin.

## Author contributions

LZ: conceptualization, methodology, validation, formal analysis, data curation, writing—original draft, writing—review and editing, visualization, funding acquisition, and guidance—original draft. WZ: writing advice. Both authors contributed to the article and approved the submitted version.
