# Supplementary material for: Impacts of family environment on adolescents’ academic achievement: The role of peer interaction quality and educational expectation gap
Source: Front Psychol. 2022 Sep 12;13:911959. doi: 10.3389/fpsyg.2022.911959 (PMC9510845; doi:10.3389/fpsyg.2022.911959)
Supplement: Supplementary file 1 [file Data_Sheet_1.zip › Revised_911959_Data and Methods/1_Methods.doc]

use "C:\Users\16634\Desktop\1_Data.dta", clear

**(1) Variables descriptive statistical / **Table 1****

sum c_sex c_age c_dusheng c_huji c_jiaoyu c_dangyuan paiming xingzhi ///

quwei huanjing qwc1 tbzl112 yyy100 yy100

**(2) Class differences, correlation analysis / **Figure 2****

pwcorr yy100 huanjing tbzl112 qwc1, star(0.05)

gr box yy100, over(c_huji) ytitle(academic achievement) intensity(0) medtype(line)

gr box yy100, over(c_jiaoyu) ytitle(academic achievement) intensity(0) medtype(line)

gr box yy100, over(c_dangyuan) ytitle(academic achievement) intensity(0) medtype(line)

gr box yy100, over(c_huji) over(c_jiaoyu) over(c_dangyuan) ytitle(Academic achievement) intensity(0) medtype(line)

**(3) Fixed effect test / **Figure 3****

twoway (scatter yy100 huanjing) (lfit yy100 huanjing , sort lpatt(solid)) if schids<=20, ///

by(schids, compact legend(off) cols(4)) xtitle(huanjing) ytitle(yy100) ysize(2) xsize(2)

**(4) Multi-level regression estimation of students' academic achievement / **Table 2****

xtset schids

global var c_sex c_age c_dusheng c_huji c_jiaoyu c_dangyuan paiming xingzhi quwei

xtreg yy100 , i(schids) mle

xtreg yy100 $var c.huanjing, i(schids) mle

xtreg yy100 $var tbzl112, i(schids) mle

xtreg yy100 $var huanjing tbzl112 qwc1, i(schids) mle

xtreg yw1100 $var huanjing tbzl112 qwc1, i(schids) mle

**(5) A Moderated mediation model test / **Table 3****

xtreg yy100 $var huanjing qwc1, i(schids) mle

xtreg yy100 $var c.huanjing##i.qwc1, i(schids) mle

xtreg tbzl112 $var huanjing qwc1, i(schids) mle

xtreg tbzl112 $var c.huanjing##i.qwc1, i(schids) mle

xtreg yy100 $var c.huanjing##i.qwc1 tbzl112, i(schids) mle

**(6) KHB test and Bootstrap 1000 test

Find it khb

khb	xtreg yy100 $var huanjing || tbzl112 , dis s

bootstrap _b[Diff], reps(1000): khb xtreg yy100 (huanjing $var) (tbzl112)
